# Common and distinct features of mammary tumors driven by Pten-deletion or activating Pik3ca mutation

**DOI:** 10.18632/oncotarget.6985

**Published:** 2016-01-22

**Authors:** Jeff C. Liu, Dong-Yu Wang, Sean E. Egan, Eldad Zacksenhaus

**Affiliations:** ^1^ Division of Advanced Diagnostics, Toronto General Research Institute - University Health Network, Toronto, Ontario, Canada; ^2^ Princess Margaret Cancer Center, Toronto, Ontario, Canada; ^3^ Campbell Family Institute for Breast Cancer Research, Princess Margaret Hospital, Toronto, Ontario, Canada; ^4^ Program in Developmental and Stem Cell Biology, The Hospital for Sick Children, Toronto, Ontario, Canada; ^5^ Department of Molecular Genetics, University of Toronto, Toronto, Ontario, Canada; ^6^ Department of Medicine, University of Toronto, Toronto, Ontario, Canada

**Keywords:** PTEN, PIK3CA, breast cancer, bioinformatics, mouse models

## Abstract

PTEN loss and PIK3CA activation both promote the accumulation of phosphatidylinositol (3, 4, 5)-trisphosphate (PIP3). While these proteins also have distinct biochemical functions, beyond the regulation of PIP3, little is known about the consequences of these differences *in vivo*. Here, we directly compared cancer signalling in mammary tumors from MMTV-Cre:Pten^f/f^ and MMTV-Cre:Pik3ca^LSL-H1047R^ mice. Using unsupervised hierarchical clustering we found that whereas MMTV-Cre:Pik3ca^LSL-H1047R^-derived tumors fall into two separate groups, designated squamous-like^Ex^ and class14^Ex^, MMTV-Cre:Pten^f/f^ tumors cluster as one group together with PIK3CA^H1047R^ class14^Ex^, exhibiting a ‘luminal’ expression profile. Gene Set Enrichment Analysis (GSEA) of Pten^Δ^ and PIK3CA^H1047R^ class14^Ex^ tumors revealed very similar profiles of signalling pathways as well as some interesting differences. Analysis of 18 signalling signatures revealed that PI3K signalling is significantly induced whereas EGFR signalling is significantly reduced in Pten^Δ^ versus PIK3CA^H1047R^ tumors. Thus, Pten^Δ^ and PIK3CA^H1047R^ tumors exhibit discernable differences that may impact tumorigenesis and response to therapy.

## INTRODUCTION

The phosphatidylinositol 3-kinase (PI3K) pathway is often induced in breast cancer through loss of the tumor suppressor Phosphatase and TENsin homolog (PTEN) or through activating mutations in PIK3CA, the catalytic subunit of PI3K [1-3]. A large body of evidence points to similar as well as distinct effects of Pten loss *versus* PIK3CA mutations on cancer progression and response to therapy [3-7]. Yet, a direct comparison of closely related tumors driven by alterations in these genes in a biologically relevant system is lacking.

Our laboratories have generated mice that develop mammary tumors following conditional Pten deletion or PIK3CA^H1047R^ mutation each induced by the same MMTV-Cre transgenic line [8, 9]. Pten^Δ^ tumors from MMTV-Cre:Pten^f/f^ mice as well as from WAP-Cre:Pten^f/f^ mice grouped closely with certain Wnt-Brca1-p53 tumors and with normal-like tumors, whereas PIK3CA^H1047R^ tumors were classified as two subtypes: squamous-like^Ex^, and class14^Ex^ with a ‘luminal’ expression profile [9, 10]. Here we used cluster analysis, Gene set enrichment analysis and pathway activity analysis to compare and contrast Pten^Δ^ and PIK3CA^H1047R^ mammary tumors. Remarkably, we found that Pten^Δ^ tumors cluster only with class14^Ex^ but not with squamous-like^Ex^ PIK3CA^H1047R^ tumors. Furthermore, class14^Ex^ tumors from both models, while substantially similar in gene set expression and multiple signalling pathways, show important differences: Pten^Δ^ tumors exhibit high PI3K signalling activity, whereas PIK3CA^H1047R^ tumors have elevated EGFR signalling. These differences may underlie tumor progression and response to therapy in Pten^Δ^ *versus* PIK3CA^H1047R^ breast cancers.

## RESULTS

### Cluster analysis reveals that Pten^Δ^ mammary tumors group together with class14^Ex^ PIK3CA^H1047R^ mammary tumors

**Figure 1:**
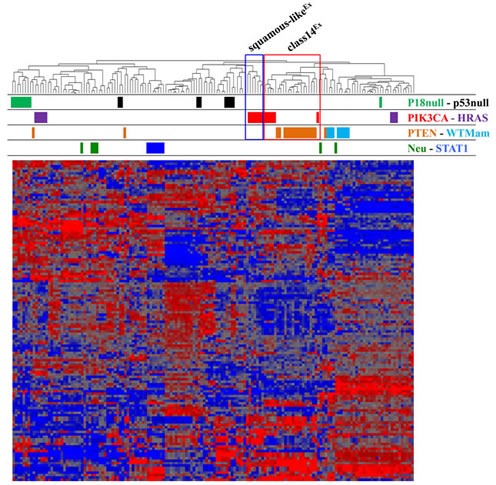
Unsupervised cluster analysis of Pten^Δ^ and PIK3CA^H1047R^ tumors with other mouse models of breast cancer (Ref. [10]) Most Pten^Δ^ tumors - derived from both MMTV-Cre:Pten^f/f^ and WAP-Cre:Pten^f/f^ mice - clustered with ~half of PIK3CA^H1047R^ tumors previously classified as class14^Ex^ (red box). The cluster of PIK3CA^H1047R^ tumors immediately left to the red box represents squamous-like^Ex^ tumors (blue box).

To compare Pten^Δ^ and PIK3CA^H1047R^ mammary tumors by RNA profiling, we integrated microarray data from Pten^Δ^ tumors with the classifier in Ref. [10], which includes PIK3CA^H1047R^ mammary tumors, using Distance Weighted Discrimination (DWD) algorithm as described [9]. As expected, unsupervised hierarchical clustering grouped PIK3CA^H1047R^ tumors on two separate leafs denoted squamous-like^Ex^ and class14^Ex^ (Figure 1). Strikingly, most Pten^Δ^ (15/18) tumors clustered closely with the class14^Ex^ subset (5/12) (Figure 1; red box), and none clustered closely with the squamous-like^Ex^ PIK3CA^H1047R^ tumors (Figure 1; blue box), indicating that at least using this classifier, PIK3CA^H1047R^ tumors show greater molecular diversity than Pten^Δ^ tumors.

### GSEA analysis demonstrates enrichment of highly similar as well as unique gene sets in class14^Ex^ Pten^Δ^ and PIK3CA^H1047R^ mammary tumors

**Figure 2:**
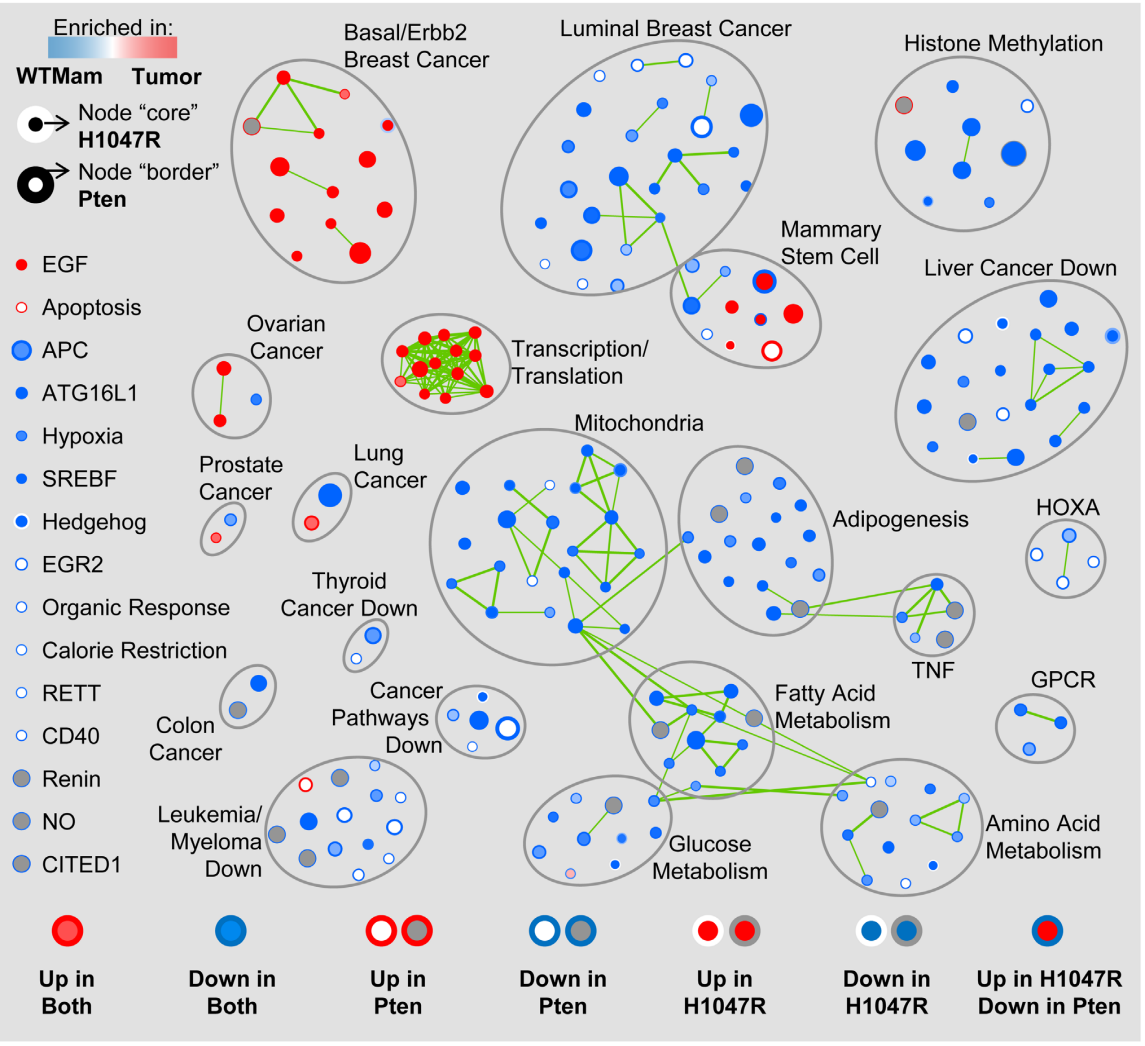
Gene Set Enrichment Analysis (GSEA) map of Pten^Δ^ and PIK3CA^H1047R^-driven class14^Ex^ tumors Only tumors from MMTV-Cre:Pten^f/f^ and MMTV-Cre:Pik3ca^LSL-H1047R^ mice grouped together in Figure 1 (red box) were used in this analysis. Circles represent pathways/nodes; clusters of nodes are grouped together; green lines connecting two or more pathways/nodes in a cluster reflect shared genes.

To identify shared and unique biological pathways that are significantly altered in tumors from MMTV-Cre:Pten^f/f^ and MMTV-Cre:Pik3ca^LSL-H1047R^ mice, we performed Gene Set Enrichment Analysis (GSEA) on those that clustered together (Figure 1, red box). We first compared each tumor type to control mammary glands analyzed on the same platform, and then identified pathways that were induced or repressed in each group relative to controls. Remarkably, most altered pathways were comparably induced (red) or repressed (blue) in both Pten^Δ^ and PIK3CA^H1047R^-driven tumors (Figure 2). These included Basal/Erbb2 Breast Cancer and Ovarian Cancer, Transcription/Translation and Mammary Stem Cell pathways that were induced, and Luminal Breast Cancer, Histone Methylation, Mitochondria and other metabolic as well as Cancer Associated pathways that were repressed (Supplemental Table 1). Thus, both tumors are more basal/less luminal, and less dependent on mitochondria, glucose, fatty acid and amino-acid metabolism relative to normal mammary glands. Notably most repressed cancer pathways are designated as “repressed in cancer”, indicating that they are activated in Pten^Δ^ and PIK3CA^H1047R^ tumors.

At the high stringent False Discovery Rate (FDR) used here (*q* < 0.01), several pathways were unique, i.e. induced or suppressed only in Pten^Δ^ or only in PIK3CA^H1047R^-driven tumors; and 3 pathways were strongly induced in opposite directions: MCBRYAN_PUBERTAL_BREAST_3_4WK_UP - from the Basal/Erbb2 cluster, and LIM_MAMMARY_LUMINAL_MATURE_DN and LIM_MAMMARY_STEM_CELL_UP from the Mammary Stem Cell cluster (Table 1; Supplemental Table 1). Overall, of 219 significant pathways in Pten^Δ^ and PIK3CA^H1047R^-driven tumors, 194 showed the same trend, 20 were unique (with NA/not applicable in one of the columns in Supplemental Table 1), and 3 were strongly contrasting. At lower stringency, *p* < 0.05, 11 additional pathways in the opposing directions were observed (Table 1).

**Table 1:** Significantly opposing pathways by GSEA

Significantly Opposing Pathways	PIK3CA-H1047R	PTEN-Loss
**MCBRYAN_PUBERTAL_BREAST_3_4WK_UP**	**2.22**	**−1.32**
**LIM_MAMMARY_LUMINAL_MATURE_DOWN**	**1.55**	**−1.9**
**LIM_MAMMARY_STEM_CELL_UP**	**1.31**	**−2**
LOPES_METHYLATED_IN_COLON_CANCER_DOWN	**1.8**	**−1.66**
CHEMOKINE_ACTIVITY	**1.74**	**−1.56**
CHEMOKINE_RECEPTOR_BINDING	**1.66**	**−1.61**
LIU_PROSTATE_CANCER_DOWN	**1.57**	**−1.69**
KEGG_CYTOKINE_CYTOKINE_RECEPTOR_INTERACTION	**1.4**	**−1.61**
G_PROTEIN_COUPLED_RECEPTOR_BINDING	**1.45**	**−1.51**
BHAT_ESR1_TARGETS_VIA_AKT1_UP	**1.31**	**−1.42**
KARLSSON_TGFB1_TARGETS_DN	**1.31**	**−1.44**
BOYLAN_MULTIPLE_MYELOMA_C_D_DOWN	**1.22**	**−1.53**
REN_ALVEOLAR_RHABDOMYOSARCOMA_DOWN	**1.18**	**−1.41**
BIOCARTA_INTRINSIC_PATHWAY	**−1.48**	**1.64**

FDR q-value<0.01; P-value<0.05; Up/Down

### Pathway activity analysis reveals a significant increase in PI3K signalling in Pten^Δ^ mammary tumors and an increase in EGFR signalling in PIK3CA^H1047R^ class14Ex tumors

**Figure 3:**
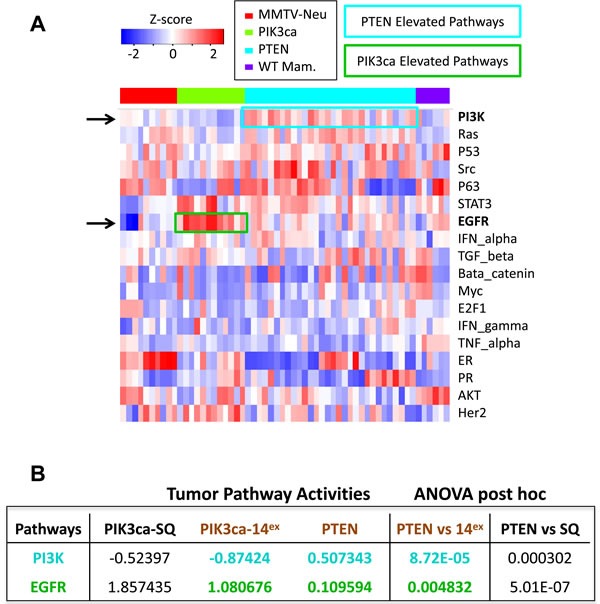
Pathway activity analysis of 18 oncogenic signalling in Pten^Δ^ and PIK3CA^H1047R^-driven class14^Ex^ tumors **A.** Pathway activities of individual samples were calculated as described by Gatza *et al*. [11]. Heat map of the 18 signalling pathways in Pten^Δ^ and PIK3CA^H1047R^-tumors as well as control normal mammary glands and MMTV-Her2/Neu tumors. The differentially expressed PI3K and EGFR pathways are highlighted. **B.** ANOVA with Tukey post hoc analysis of Pten^Δ^ tumors and the 2 subtypes of PIK3CA^H1047R^ tumors, revealing a significant increase in PI3K pathway signature in Pten^Δ^ tumors, and a significant increase in EGFR signalling in PIK3CA^H1047R^ tumors.

To further dissect differences between Pten^Δ^ and PIK3CA^H1047R^ tumors, we determined signature activities for all 18 signalling pathways described by Gatza *et al*. [11]. Strikingly, only PI3K and EGFR pathways were significantly altered: the PI3K pathway was induced in Pten^Δ^ relative to PIK3CA^H1047R^ tumors (*P* = 8.72E-05 by ANOVA), while EGFR signaling was induced in PIK3CA^H1047R^- *versus* Pten^Δ^-tumors (*P* = 0.0048; Figure 3). These differences were not only seen when comparing Pten^Δ^ to PIK3CA^H1047R^ class14^Ex^ tumors, but also when comparing Pten^Δ^ to PIK3CA^H1047R^ squamous-like^Ex^ lesions (Figure 3B).

### mRNA expression of secreted factors involved in EGFR signalling is similar in Pten^Δ^ and PIK3CA^H1047R^-tumors

**Figure 4:**
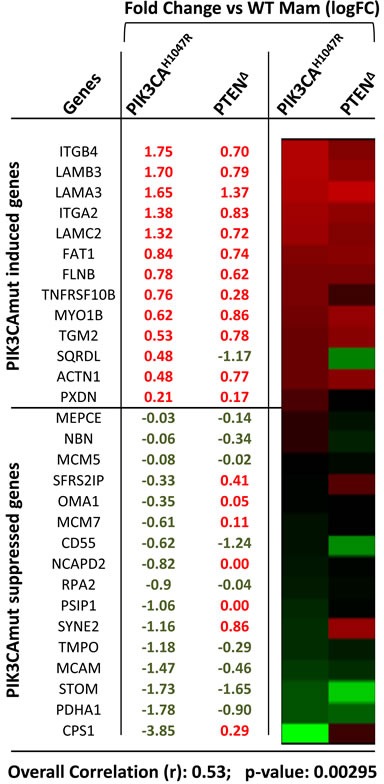
Relative expression (values and heat map) and correlation analysis of PI3KCA-regulated genes associated with EGFR activation in Pten^Δ^ and PIK3CA^H1047R^ mammary tumors

Multiple mechanisms may account for the observed differences in PI3K and EGFR signalling between Pten^Δ^ and PIK3CA^H1047R^-tumors (Discussion). One possibility is suggested by a recent observation by the Arteaga group, that activating PIK3CA mutations (E545K and H1047R) in human breast cell lines alter expression of secreted proteins, cell surface receptors and ECM-interacting proteins leading to induction of EGFR signaling [12]. Of these proteins, 72 were detected in both E545K- and H1047R-over-expressing cell lines relative to controls (43 upregulated and 29 down-regulated), and a fraction of these were up- or down-regulated at the mRNA level [12]. We asked whether these genes are also similarly up- or down-regulated in Pten^Δ^ tumors, or whether elevated autocrine/paracrine EGFR signalling is unique to PIK3CA-driven tumors. We therefore compared the expression of these genes in mouse Pten^Δ^ *versus* PIK3CA^H1047R^-class14^Ex^ mammary tumors. In the mouse data set, 13 genes were upregulated and 16 were downregulated in the same direction as the human PIK3CA-altered proteins. Remarkably, 12 of 13 upregulated genes in PIK3CA^H1047R^-tumors including integrins (ITGB4, ITGA2), laminins (LAMA3, LAMC2), FAT Tumor Suppressor Homolog 1, and peroxidasin (PXDN) were upregulated, and 9 of 16 were down-regulated in Pten^Δ^ tumors, with overall correlation of 0.53 (*P* = 0.000295) (Figure 4). While proteomic analysis is required to confirm these observations, our results suggest that Pten loss may induce EGFR signalling as seen in human PIK3CA tumors, and therefore may not explain the differences between PIK3CA mutant and PTEN-deficient tumors.

## DISCUSSION

We report that Pten-loss driven mammary tumors exhibit elevated PI3K pathway activity and reduced EGFR activity compared to PIK3CA mutant tumors. In concordance with these findings, a recent analysis of invasive lobular adenocarcinomas has demonstrated that PI3K/AKT signalling is more highly elevated in PTEN-deficient tumors than in PIK3CA mutants [13]. Thus, within closely related tumors driven by alterations in these genes, PTEN-loss has overtly distinct effects on cancer signalling compared to PI3K mutations. In contrast, analysis of divergent breast cancer cell lines revealed that mutations in PIK3CA but not PTEN loss coincide with increased sensitivity to mTOR inhibitors [2]. The latter report on the opposite differential sensitivity may reflect the different breast cancer subtypes in which PIK3CA mutations and PTEN loss usually occur (luminal vs. basal-like, respectively), and the effect of cooperating oncogenic signalling on PI3K/PKB-AKT/mTOR activity in the different subtypes of breast cancer. In contrast, both our results and the abovementioned study (Ref. [13]) analyzed the effects of PIK3CA mutations *versus* PTEN loss in closely related breast cancer subtypes, and the differences observed may truly reflect different effects these genes have on PI3K pathway activity.

A multitude of possibilities may underlie the observed differences in pathway activation. Likely, the differences reflect inherent differences in the effects of PTEN-loss vs PIK3CA mutation on breast epithelial cells. Indeed, while these proteins have opposing effects on phosphatidylinositol (3, 4, 5)-trisphosphate signaling, they also have additional unique functions. For example, PTEN can dephosphorylate certain proteins, and can enter and function within the nucleus in a phosphatase-independent manner or be secreted from cells to affect their neighbors [14-19]. A future challenge would be to define the unique functions of PIK3CA and PTEN underlying their differential affects on EGFR and PI3K signaling.

In conclusions, our results demonstrate that PIK3CA^H1047R^ induces two molecularly distinct tumor subtypes: class14^Ex^, which is also induced by Pten-loss, and squamous-like^Ex^, which is uniquely induced by PIK3CA^H1047R^. Within the closely related Pten^Δ^ and PIK3CA^H1047R^ class14^Ex^ tumors, GSEA analysis revealed that most enriched gene sets are shared by the two tumor types, yet, some differences were noted. Using pathway signature analysis of 18 oncogenic signalling, we found that PI3K and EGFR signalling were significantly different in Pten^Δ^ vs. PIK3CA^H1047R^- tumors. These pathways may account for differences in drug sensitivity between Pten^Δ^ vs. PIK3CA^H1047R^ tumors [5]. The different response to therapy may also reflect different oncogenic events that cooperate with each gene, leading to distinct tumor subtypes in the course of cancer progression. The latter possibility is illustrated by squamous-like^Ex^ tumors observed in PIK3CA^H1047R^ but not Pten^Δ^ lesions (Figure 1). Moreover, in human breast cancer, PTEN-loss is most frequent in basal-like breast cancer, whereas the spectrum of PI3K mutation is wider and most frequently observed in luminal tumors [20]. Therapeutic strategies should therefore be designed with the recognition that oncogenic signalling through PIK3CA-mutation and PTEN-loss are distinct, and should be based on comprehensive mutational analysis, tumor subtyping, identification of cooperating oncogenic events and ultimately direct assessment of tumor response to anti-PI3K/AKT/mTOR and anti-RAS/MAPK therapies.

## MATERIALS AND METHODS

### Tumor samples and microarray analysis

Microarray analysis with PTEN^Δ^ mouse tumor models (GSE39955) and wild-type mammary glands (GSE62016) were carried out using Affymetrix Mouse Gene 1.0 ST. Microarray data were normalized using RMA method via Partek software, and log2-transformed gene expression values were obtained. Normalized expression data for tumors with PIK3CA^H1047R^ mutation and corresponding wild-type mammary glands were downloaded from GSE42640.

### Molecular subtype classification

“Distance Weighted Discrimination” (DWD) was used to integrate data from the mouse models with a reference dataset containing human breast cancer subtypes pre-determined by PAM50 (GSE18229). Unsupervised hierarchical clustering (complete linkage) with the intrinsic genes signature (Herschkowitz et al, 2007) was used to group tumor samples for subtype classification.

### Gene set enrichment analysis

Differential gene expressions comparing: 1) PIK3CA^H1047R^ vs WT mammary glands; and 2) PTEN^Δ^ vs WT mammary glands; were calculated by Moderate T test using limma package in R. Genes were ordered using the t-values corresponding to each pair-wise comparison as rank files for Gene Set Enrichment Analysis (GSEA, [21] with 1000 permutations and gene-sets size between 8 and 500. The gene-sets included in the GSEA analyses were obtained from KEGG, MsigDB-c2, NCI, Biocarta, IOB, Netpath, Human Cyc, Reactome and the Gene Ontology (GO) databases, updated March 2012 (http://baderlab.org/GeneSets). An enrichment map (version 1.2 of Enrichment Map software [22] was generated for each comparison using enriched gene-sets with a nominal *p*-value <0.05 and the overlap coefficient set to 0.5 as described [23, 24].

### Pathway activities

Pathway activities of individual samples were calculated from microarray data as described [11], and normalized by z-score. Heat map was generated by the function heatmap.2 in gplots package in R. ANOVA with Tukey post hoc was used to determine the significance of differences between Pten^Δ^ tumors and the 2 subtypes of PIK3CA^H1047R^ tumors, squamous-like and class14^EX^.

### Statistic analysis

Correlation analysis was performed comparing logFC (log2 of fold change) values of PIK3CA^H1047R^ vs WT mammary glands with values of PTEN^Δ^ vs WT mammary glands by the function corr.test in psych package in R that calculates both the correlation coefficient (r) and significance (*p*-value).

## SUPPLEMENTARY MATERIAL TABLE


