# Supplementary material for: Common and distinct features of mammary tumors driven by Pten-deletion or activating Pik3ca mutation
Source: Oncotarget. 2016 Jan 22;7(8):9060–8. doi: 10.18632/oncotarget.6985 (PMC4891026; doi:10.18632/oncotarget.6985)
Supplement: Supplementary file 1 [file oncotarget-07-9060-s001.pdf]

Common and distinct features of mammary tumors driven by Pten-deletion or activating Pik3ca mutation

Supplementary Material

Supplemental Table S1: Pathways enriched in MMTV-Cre:Pten$^{f/f}$- and MMTV-Cre:Pik3ca$^{LSL\text{-}H1047R}$-driven tumors as determined by GSEA (Fig. 2).

P-value < 0.01 Q-value < 0.01

	H1047R	PTEN
**Basal/Erbb2 Breast Cancer**		
LANDIS_ERBB2_BREAST_TUMORS_324_UP	2.55	3.12
LANDIS_BREAST_CANCER_PROGRESSION_UP	2.53	2.72
COLDREN_GEFITINIB_RESISTANCE_DN	2.63	2.7
CHARAFE_BREAST_CANCER_BASAL_VS_MESENCHYMAL_UP	2.16	2.63
MCBRYAN_PUBERTAL_BREAST_4_5WK_UP	3.24	2.54
LANDIS_ERBB2_BREAST_TUMORS_65_UP	2	2.39
AIGNER_ZEB1_TARGETS	2.15	2.38
LANDIS_ERBB2_BREAST_PRENEOPLASTIC_UP	NA	2.22
CHARAFE_BREAST_CANCER_LUMINAL_VS_MESENCHYMAL_UP	2.36	2.18
JAEGER_METASTASIS_DN	1.69	2.14
ONDER_CDH1_TARGETS_2_DN	1.98	2.08
PID_ECADHERIN_STABILIZATION_PATHWAY	1.75	2.07
MCBRYAN_PUBERTAL_BREAST_3_4WK_UP	2.22	-1.32
**Luminal Breast Cancer**		
LANDIS_ERBB2_BREAST_TUMORS_324_DN	-1.87	-2.88
NAKAYAMA_SOFT_TISSUE_TUMORS_PCA2_DN	-1.83	-2.71
LANDIS_ERBB2_BREAST_PRENEOPLASTIC_DN	-1.92	-2.7
LANDIS_BREAST_CANCER_PROGRESSION_DN	-1.72	-2.67
SMID_BREAST_CANCER_LUMINAL_A_UP	-1.64	-2.61
MCBRYAN_PUBERTAL_BREAST_4_5WK_DN	-2.03	-2.51
LANDIS_ERBB2_BREAST_TUMORS_65_DN	-1.63	-2.48
SMID_BREAST_CANCER_NORMAL_LIKE_UP	-1.38	-2.35
SCHUETZ_BREAST_CANCER_DUCTAL_INVASIVE_UP	-1.4	-2.31
MCBRYAN_PUBERTAL_BREAST_6_7WK_UP	-1.03	-2.25
LINDGREN_BLADDER_CANCER_CLUSTER_2B	-1.3	-2.22
BERTUCCI_INVASIVE_CARCINOMA_DUCTAL_VS_LOBULAR_DN	-1.89	-2.17
HOWLIN_PUBERTAL_MAMMARY_GLAND	-1.17	-2.16
SMID_BREAST_CANCER_RELAPSE_IN_LUNG_DN	-1.57	-2.14
NIELSEN_GIST_AND_SYNOVIAL_SARCOMA_DN	-1.26	-2.11
MCBRYAN_PUBERTAL_BREAST_5_6WK_DN	-1.22	-2.09
NAKAYAMA_SOFT_TISSUE_TUMORS_PCA1_UP	-1.21	-2.07
CHARAFE_BREAST_CANCER_BASAL_VS_MESENCHYMAL_DN	-1.37	-2.07
ONDER_CDH1_TARGETS_2_UP	-1.48	-2.06
ANASTASSIOU_CANCER_MESENCHYMAL_TRANSITION_SIGNATURE	-1.43	-2.05
DURAND_STROMA_S_UP	-1.53	-2.05
GOZGIT_ESR1_TARGETS_UP	-1.61	-2.05
DAVICIONI_MOLECULAR_ARMS_VS_ERMS_DN	-1.31	-1.99
CHARAFE_BREAST_CANCER_LUMINAL_VS_MESENCHYMAL_DN	-1.13	-1.95
FARMER_BREAST_CANCER_CLUSTER_5	-1.64	-1.91
**Transcription/Translation**		
KEGG_RIBOSOME	2.11	2.7
REACTOME_PEPTIDE_CHAIN_ELONGATION	2.12	2.68
REACTOME_SRP_DEPENDENT_COTRANSLATIONAL_PROTEIN_TARGETING_TO_MEMBRANE	2.07	2.64
REACTOME_INFLUENZA_LIFE_CYCLE	1.6	2.59
REACTOME_3_UTR_MEDIATED_TRANSLATIONAL_REGULATION	2.22	2.54
REACTOME_INFLUENZA_VIRAL_RNA_TRANSCRIPTION_AND_REPLICATION	1.91	2.53
REACTOME_TRANSLATION	1.77	2.37
REACTOME_NONSENSE_MEDIATED_DECAY_ENHANCED_BY_THE_EXON_JUNCTION_COMPLEX	1.9	2.32
BILANGES_SERUM_AND_RAPAMYCIN_SENSITIVE_GENES	1.63	2.26
REACTOME_FORMATION_OF_THE_TERNARY_COMPLEX_AND_SUBSEQUENTLY_THE_43S_COMPLEX	1.8	2.14
STRUCTURAL_CONSTITUENT_OF_RIBOSOME	1.81	2.12
CHNG_MULTIPLE_MYELOMA_HYPERPLOID_UP	1.92	2.1
REACTOME_METABOLISM_OF_RNA	1.66	2.07
**Mammary Stem Cell**		
ZHANG_BREAST_CANCER_PROGENITORS_UP	1.16	2.23
PECE_MAMMARY_STEM_CELL_UP	1.78	2.12
LIM_MAMMARY_STEM_CELL_DN	2.23	2
LEE_NEURAL_CREST_STEM_CELL_UP	-1.31	-1.98
BOQUEST_STEM_CELL_CULTURED_VS_FRESH_DN	-1.51	-2.04
RAMALHO_STEMNESS_DN	-1.32	-2.1
BOQUEST_STEM_CELL_UP	-1.4	-2.48

FRIDMAN_IMMORTALIZATION_DN	2.29	-0.95

Pathway Clusters

Mitochondria Function	H1047R	PTEN
P-value < 0.01 Q-value < 0.01		
GENERATION_OF_PRECURSOR_METABOLITES_AND_ENERGY	-1.65	-2.22
KEGG_CITRATE_CYCLE_TCA_CYCLE	-1.89	-2.15
KEGG_PEROXISOME	-1.79	-2.07
REACTOME_PHASE1_FUNCTIONALIZATION_OF_COMPOUNDS	-1.76	-2.07
REACTOME_CYTOCHROME_P450_ARRANGED_BY_SUBSTRATE_TYPE	-1.79	-2
REACTOME_PYRUVATE_METABOLISM_AND_CITRIC_ACID_TCA_CYCLE	-1.85	-1.99
COFACTOR_BINDING	-0.72	-1.98
KEGG_METABOLISM_OF_XENOBIOTICS_BY_CYTOCHROME_P450	-1.41	-1.95
REACTOME_BIOLOGICAL_OXIDATIONS	-1.79	-1.92
KEGG_PYRUVATE_METABOLISM	-1.45	-1.92
REACTOME_CITRIC_ACID_CYCLE_TCA_CYCLE	-1.8	-1.91
MITOCHONDRIAL_PART	-1.71	-1.91
OXIDOREDUCTASE_ACTIVITY	-1.68	-1.89
ELECTRON_TRANSPORT_GO_0006118	-1.21	-1.89
REACTOME_TCA_CYCLE_AND_RESPIRATORY_ELECTRON_TRANSPORT	-2.09	-1.88
REACTOME_RESPIRATORY_ELECTRON_TRANSPORT	-1.99	-1.52
KEGG_PARKINSONS_DISEASE	-2.02	-1.45
INORGANIC_CATION_TRANSMEMBRANE_TRANSPORTER_ACTIVITY	-1.91	-0.99
REACTOME_RESPIRATORY_ELECTRON_TRANSPORT_ATP_SYNTHESIS_BY_CHEMIOSMOTIC_COUPLING_AND_HEAT_PRODUCTION_BY_UNCOUPLING_PROTEINS_	-1.95	-1.55

Fatty Acid Metabolism		
REACTOME_TRIGLYCERIDE_BIOSYNTHESIS	-1.72	-2.33
LIPID_CATABOLIC_PROCESS	-1.86	-2.14
KEGG_FATTY_ACID_METABOLISM	-2.02	-2.14
REACTOME_FATTY_ACID_TRIACYLGLYCEROL_AND_KETONE_BODY_METABOLISM	-1.81	-2.12
CARBOXYLESTERASE_ACTIVITY	-1.62	-2.09
CELLULAR_LIPID_CATABOLIC_PROCESS	-1.84	-2.07
CARBOXYLIC_ACID_METABOLIC_PROCESS	-1.95	-2
REACTOME_FATTY_ACYL_COA_BIOSYNTHESIS	NA	-2
ORGANIC_ACID_METABOLIC_PROCESS	-1.9	-2
ENERGY_DERIVATION_BY_OXIDATION_OF_ORGANIC_COMPOUNDS	-1.92	-1.96
KEGG_BIOSYNTHESIS_OF_UNSATURATED_FATTY_ACIDS	NA	-1.95
MOOTHA_FFA_OXYDATION	-1.94	-1.92
ORGANIC_ACID_TRANSPORT	-1.43	-1.91
LIPID_METABOLIC_PROCESS	-1.7	-1.9
KEGG_LINOLEIC_ACID_METABOLISM	-1.55	-1.9
MONOCARBOXYLIC_ACID_METABOLIC_PROCESS	-1.97	-1.89
CARBOXYLIC_ACID_TRANSPORT	-1.44	-1.89

Adipogenesis		
BURTON_ADIPOGENESIS_6	-2.03	-2.81
WANG_CLASSIC_ADIPOGENIC_TARGETS_OF_PPARG	-2.03	-2.62
WAKABAYASHI_ADIPOGENESIS_PPARG_RXRA_BOUND_WITH_H4K20ME1_MARK	-1.78	-2.6
NADLER_OBESITY_DN	-1.58	-2.5
GERHOLD_ADIPOGENESIS_UP	-1.98	-2.48
STEGER_ADIPOGENESIS_UP	-2.07	-2.47
LI_ADIPOGENESIS_BY_ACTIVATED_PPARG	NA	-2.46
VERNOCHET_ADIPOGENESIS	NA	-2.33
BURTON_ADIPOGENESIS_5	-2.02	-2.29
KEGG_ADIPOCYTOKINE_SIGNALING_PATHWAY	-1.71	-2.17
URS_ADIPOCYTE_DIFFERENTIATION_UP	-2.01	-2.16
NAKAMURA_ADIPOGENESIS_LATE_UP	-1.62	-2.15
NIELSEN_LIPOSARCOMA_UP	NA	-2.15
WAKABAYASHI_ADIPOGENESIS_PPARG_RXRA_BOUND_36HR	-1.46	-2.12
REACTOME_TRANSCRIPTIONAL_REGULATION_OF_WHITE_ADIPOCYTE_DIFFERENTIATION	-1.67	-2.06
IZADPANAH_STEM_CELL_ADIPOSE_VS_BONE_DN	-1.68	-1.94
URS_ADIPOCYTE_DIFFERENTIATION_DN	-1.53	-1.93

Glucose Metabolism		
KEGG_INSULIN_SIGNALING_PATHWAY	-1.44	-2.17

REACTOME_GLUCOSE_METABOLISM	-1.57	-2.03
BIOCARTA_CHREBP2_PATHWAY	-1.38	-1.99
TSENG_IRS1_TARGETS_DN	-1.56	-1.96
MOOTHA_GLYCOGEN_METABOLISM	NA	-1.94
REACTOME_REGULATION_OF_BETA_CELL_DEVELOPMENT	-1.78	-1.91
APPEL_IMATINIB_RESPONSE	1.38	-1.9
REACTOME_GLYCEROPHOSPHOLIPID_BIOSYNTHESIS	-1.5	-1.9
GLUCOSE_METABOLIC_PROCESS	-1.88	-1.53
DIGESTION	-1.99	-0.94

Pathway Clusters

P-value < 0.01 Q-value < 0.01

Liver Cancer Down	H1047R	PTEN
LEE_LIVER_CANCER_MYC_E2F1_DN	-2.09	-2.5
LEE_LIVER_CANCER_E2F1_DN	-1.97	-2.35
LEE_LIVER_CANCER_MYC_TGFA_DN	-2.03	-2.28
KEEN_RESPONSE_TO_ROSIGLITAZONE_UP	-1.65	-2.25
LEE_LIVER_CANCER_MYC_DN	-1.97	-2.21
CAIRO_HEPATOBLASTOMA_CLASSES_DN	-1.16	-2.17
HSIAO_LIVER_SPECIFIC_GENES	-1.92	-2.11
LEE_LIVER_CANCER_CIPROFIBRATE_DN	-2.07	-2.08
CHIANG_LIVER_CANCER_SUBCLASS_PROLIFERATION_DN	-1.88	-2.07
CAIRO_HEPATOBLASTOMA_POOR_SURVIVAL	NA	-2.04
WENG_POR_TARGETS_LIVER_UP	-1.95	-2.03
WOO_LIVER_CANCER_RECURRENCE_DN	-1.66	-2.01
LEE_LIVER_CANCER_DENA_DN	-1.96	-2.01
LEE_LIVER_CANCER_SURVIVAL_UP	-1.82	-2
SERVITJA_LIVER_HNF1A_TARGETS_UP	-1.21	-1.98
SHETH_LIVER_CANCER_VS_TXNIP_LOSS_PAM4	-1.68	-1.95
CHIANG_LIVER_CANCER_SUBCLASS_CTNNB1_UP	-1.73	-1.89
ACEVEDO_LIVER_CANCER_WITH_H3K27ME3_UP	-1.88	-1.32
OHGUCHI_LIVER_HNF4A_TARGETS_DN	-2	-1.28
SU_LIVER	-1.9	-0.46
**Ovarian Cancer**		
WAMUNYOKOLI_OVARIAN_CANCER_GRADES_1_2_UP	2.02	2.12
WAMUNYOKOLI_OVARIAN_CANCER_LMP_UP	1.86	2.1
WAMUNYOKOLI_OVARIAN_CANCER_GRADES_1_2_DN	-1.53	-2.03
**Colon Cancer**		
SABATES_COLORECTAL_ADENOMA_DN	-1.96	-2.18
LOPES_METHYLATED_IN_COLON_CANCER_UP	NA	-1.94
**Cancer Pathways Down**		
WONG_ENDMETRIUM_CANCER_DN	-1.32	-2.55
VECCHI_GASTRIC_CANCER_EARLY_DN	-1.78	-2.1
DAIRKEE_CANCER_PRONE_RESPONSE_E2	-0.93	-2.09
WEST_ADRENOCORTICAL_TUMOR_DN	-1.16	-1.94
RICKMAN_HEAD_AND_NECK_CANCER_C	-2.02	-1.02
**Leukemia/Myeloma Down**		
WANG_MLL_TARGETS	-1.57	-2.14
HADDAD_T_LYMPHOCYTE_AND_NK_PROGENITOR_DN	-1.6	-2.02
CORRE_MULTIPLE_MYELOMA_DN	-1.22	-2.01
TONKS_TARGETS_OF_RUNX1_RUNX1T1_FUSION_GRANULOCYTE_DN	NA	-1.97
HEMATOPOIETIN_INTERFERON_CLASSD200_DOMAIN_CYTOKINE_RECEPTOR_ACTIVITY	-0.78	-1.95
TONKS_TARGETS_OF_RUNX1_RUNX1T1_FUSION_HSC_DN	-1.37	-1.94
SEKI_INFLAMMATORY_RESPONSE_LPS_DN	NA	-1.94
BASSO_HAIRY_CELL_LEUKEMIA_DN	-1.06	-1.91
LENAOUR_DENDRITIC_CELL_MATURATION_UP	-1.44	-1.91
VERHAAK_AML_WITH_NPM1_MUTATED_DN	-1.12	-1.9
ZHAN_MULTIPLE_MYELOMA_DN	-1.35	-1.89
REACTOME_COMPLEMENT_CASCADE	NA	-1.89
PICCALUGA_ANGIOIMMUNOBLASTIC_LYMPHOMA_UP	-0.93	-1.89
ZHANG_TLX_TARGETS_36HR_DN	0.65	2.27
**Thyroid Cancer Down**		
WATTEL_AUTONOMOUS_THYROID_ADENOMA_DN	-1.2	-2.08
DELYS_THYROID_CANCER_DN	-1.34	-1.97
**Lung Cancer**		
LI_AMPLIFIED_IN_LUNG_CANCER	1.48	2.27
SWEET_LUNG_CANCER_KRAS_DN	-1.66	-2.26
**Prostate Cancer**		
GU_PDEF_TARGETS_DN	1.52	2.13
LIU_VAV3_PROSTATE_CARCINOGENESIS_UP	-1.36	-2

Pathway Clusters

Histone Methylation	P-value < 0.01	Q-value < 0.01	H1047R	PTEN
MIKKELSEN_MCV6_HCP_WITH_H3K27ME3			-1.75	-2.03
MEISSNER_NPC_HCP_WITH_H3K4ME2_AND_H3K27ME3			-1.68	-2
MIKKELSEN_IPS_WITH_HCP_H3K27ME3			-1.8	-1.97
MIKKELSEN_ES_ICP_WITH_H3K4ME3_AND_H3K27ME3			-1.17	-1.95
MIKKELSEN_NPC_HCP_WITH_H3K27ME3			-1.87	-1.95
MIKKELSEN_MEF_ICP_WITH_H3K4ME3_AND_H3K27ME3			-1.59	-1.89
ROLEF_GLIS3_TARGETS			-1.95	-1.48
LUND_SILENCED_BY_METHYLATION			NA	2.22
BENPORATH_PRC2_TARGETS			-1.94	NA
**Amino Acid Metabolism**				
KEGG_PPAR_SIGNALING_PATHWAY			-2.08	-2.57
KEGG_VALINE_LEUCINE_AND_ISOLEUCINE_DEGRADATION			-1.76	-2.24
KEGG_ARACHIDONIC_ACID_METABOLISM			-1.46	-2.22
KEGG_PROPANOATE_METABOLISM			-1.62	-2.13
KEGG_HISTIDINE_METABOLISM			-0.76	-2.01
AMINE_TRANSPORT			-1.51	-1.94
REACTOME_SYNTHESIS_OF_PA			-1.33	-1.93
REACTOME_BRANCHED_CHAIN_AMINO_ACID_CATABOLISM			NA	-1.93
REACTOME_AMINO_ACID_TRANSPORT_ACROSS_THE_PLASMA_MEMBRANE			-1.77	-1.92
**TNF**				
RUAN_RESPONSE_TO_TNF_DN			-1.9	-2.86
RUAN_RESPONSE_TO_TNF_TROGLITAZONE_DN			-1.72	-2.66
RUAN_RESPONSE_TO_TROGLITAZONE_UP			-1.5	-2.32
RUAN_RESPONSE_TO_TROGLITAZONE_DN			NA	-2.22
RUAN_RESPONSE_TO_TNF_TROGLITAZONE_UP			NA	-1.95
**GPCR**				
REACTOME_G_ALPHA_S_SIGNALLING_EVENTS			-1.4	-2.07
G_PROTEIN_SIGNALING_COUPLED_TO_CYCLIC_NUCLEOTIDE_SECOND_MESSENGER			-1.55	-2
CYCLIC_NUCLEOTIDE_MEDIATED_SIGNALING			-1.61	-1.96
**HOXA**				
TAKEDA_TARGETS_OF_NUP98_HOXA9_FUSION_8D_DN			-1.28	-2.09
TAKEDA_TARGETS_OF_NUP98_HOXA9_FUSION_16D_UP			-1.19	-2.05
YAO_HOXA10_TARGETS_VIA_PROGESTERONE_UP			-1.11	-2
TAKEDA_TARGETS_OF_NUP98_HOXA9_FUSION_10D_DN			-1.06	-2
**Other Pathways**				
REACTOME_APOPTOTIC_EXECUTION_PHASE			1.08	2.21
AMIT_EGF_RESPONSE_60_MCF10A			2.25	1.68
CADWELL_ATG16L1_TARGETS_UP			-1.95	-2.61
BASSO_CD40_SIGNALING_DN			-1.19	-2.16
SANSOM_APC_TARGETS_DN			-1.32	-2.14
KEGG_RENIN_ANGIOTENSIN_SYSTEM			NA	-2.04
HORTON_SREBF_TARGETS			-1.73	-2.03
WINTER_HYPOXIA_DN			-1.59	-2.01
RESPONSE_TO_ORGANIC_SUBSTANCE			-0.86	-2.01
LE_EGR2_TARGETS_DN			-1.1	-2.01
HOWLIN_CITED1_TARGETS_1_UP			NA	-1.94
TRAYNOR_RETT_SYNDROM_UP			-0.86	-1.91
REACTOME_NITRIC_OXIDE_STIMULATES_GUANYLATE_CYCLASE			NA	-1.89
LEE_CALORIE_RESTRICTION_MUSCLE_UP			-1.26	-1.89
YAUCH_HEDGEHOG_SIGNALING_PARACRINE_DN			-1.91	-1.24
**Contrasting Pathways**				
LIM_MAMMARY_STEM_CELL_UP			1.31	-2
LIM_MAMMARY_LUMINAL_MATURE_DN			1.55	-1.9

Pathways enriched in MMTV-Cre:Pten$^{f/f}$- and MMTV-Cre:Pik3ca$^{LSL-H1047R}$-driven tumors as determined by GSEA (Fig. 2).
Values on the right are Normalized Enrichment Scores. +/- signs reflect pathways that are induced/repressed relative to control normal glands.
Red denotes FDR<0.01; Blue indicates P<0.05, Black=not significant, NA=not applicable.
